# Real-World Experience in the Treatment of Biofilm-Associated Wounds Using Medical-Grade Honey: A Retrospective Case Series

**DOI:** 10.3390/antibiotics15020150

**Published:** 2026-02-02

**Authors:** Yun-Nan Lin

**Affiliations:** 1 Wesing Breast Hospital, Kaohsiung 813, Taiwan; yunnan1123@gmail.com; 2 Regenerative Medicine and Cell Therapy Research Center, Kaohsiung 807, Taiwan; 3 Department of Surgery, Division of Plastic Surgery, Kaohsiung Medical University Hospital, Kaohsiung 807, Taiwan

**Keywords:** medical-grade honey, biofilm, case series, wound healing

## Abstract

**Background/Objectives**: Wounds complicated by biofilm formation remain a major challenge in wound management. Medical-grade honey (MGH) possesses potent antimicrobial and biofilm-disrupting properties. This study aimed to evaluate the clinical effectiveness of MGH in the treatment of biofilm-associated wounds. **Methods**: A retrospective case series was conducted involving ten patients with biofilm-suspected wounds treated at Kaohsiung Medical University Hospital and Wesing Hospital. All wounds exhibited positive bacterial cultures and clinical signs of biofilm formation. MGH was applied topically, and wound progression was monitored throughout the treatment period. **Results**: Eight out of ten wounds achieved complete healing, with a median healing time of 16 weeks (range: 4–46 weeks). Most wounds demonstrated reduced exudate and inflammation, along with progressive granulation and epithelialization. Two wounds did not fully heal within the follow-up period. **Conclusions**: MGH appears to be a promising adjunctive therapy for wounds associated with biofilm formation, particularly in cases refractory to conventional antibiotic therapy. Further large-scale, controlled studies are warranted to confirm these preliminary findings.

## 1. Introduction

Wounds complicated by biofilms are resistant to conventional therapies and are associated with prolonged healing times [1,2]. A biofilm is an aggregate of microorganisms encased in a self-produced matrix of extracellular polymeric substances, e.g., polysaccharides, proteins, and extracellular DNA. This matrix shields the microbes from external factors, such as antibiotics and the body’s immune system, often leading to delayed healing. *Pseudomonas aeruginosa* is a common pathogen in such infections, known for its ability to form robust biofilms and produce virulence factors such as pyocyanin and elastase, which impair host immune responses and tissue regeneration [3,4,5]. Other frequently implicated bacteria include *Staphylococcus aureus*, particularly methicillin-resistant strains (MRSA), *Enterococcus faecalis*, and anaerobes such as *Bacteroides* spp. and *Prevotella* spp. [6]. These polymicrobial communities exhibit synergistic interactions, further enhancing biofilm stability and antimicrobial resistance.

Early identification and aggressive management of biofilms are essential for promoting wound healing, especially in wounds such as diabetic foot ulcers and venous leg ulcers. Biofilms are diagnosed primarily clinically, based on signs such as chronic inflammation, poor granulation, increased exudate, and failure to respond to standard care [7]. Managing wound biofilms requires a multimodal approach that targets the biofilm structure and underlying microbial load. Serial wound measurements with a reduction in exudate, slough, and bioburden, and improvement in granulation tissue are indicators of a decrease in biofilm [8,9,10].

Medical-grade honey (MGH) has been shown to possess antimicrobial properties and the ability to disrupt biofilms while promoting tissue regeneration. MGH is distinguished from regular honey by its stringent collection and production standards, ensuring it is free of contaminants and microorganisms and guaranteeing its quality, safety, and efficacy for medical applications [11]. MGH exhibits broad-spectrum antimicrobial activity through its low pH, high osmolarity, hydrogen peroxide release, and bioactive compounds. It disrupts and inhibits biofilm formation via its antimicrobial and debriding activity, enhancing wound bed preparation. Additionally, MGH promotes wound healing by maintaining a moist healing environment, reducing inflammation, and stimulating angiogenesis and reepithelialization [11,12].

MGH has been used to treat wounds in infected pressure injuries and diabetic wounds [13,14]. Though infected wounds and hard-to-heal wounds have been treated with success, data on their biofilm-related activity is limited [15,16]. Biofilm represents a major barrier to wound healing and a frequent cause of treatment failure; therefore, evaluating therapeutic modalities that can effectively manage biofilm is clinically valuable. This case series provides real-world evidence on the application of MGH in biofilm-complicated wounds and highlights its potential as a practical, accessible strategy for biofilm management in routine clinical practice. Our findings underscore the importance of MGH as a low-cost yet effective adjunct in the management of biofilm-associated wounds.

## 2. Results

This study included a total of 10 cases, with 8 patients treated at Kaohsiung Medical University Hospital (KMUH) and 2 at Wesing Hospital (WS); see Table 1. The patients had a median age of 58 years, ranging from 33 to 84 years, with an equal gender distribution of 5 males and 5 females. Wound types included 5 hard-to-heal wounds, 3 diabetic foot ulcers, and 2 mixed leg ulcers. The wound locations varied: abdomen (1 case), ankle (3), foot (1), leg (2), toe (1), and breast (2). Wound sizes averaged 3.83 ± 0.98 cm in length (range: 0.8–11.0 cm) and 1.55 ± 0.23 cm in width (range: 0.5–3.0 cm). Common underlying conditions among patients were diabetes mellitus (6 cases), heart disease (3), peripheral arterial disease (2), uremia (2), cancer (2 cases involving breast or oral cancer), and chronic venous insufficiency (2). In terms of cancer treatment, 7 patients had no history of radiotherapy or chemotherapy, while 2 had received chemotherapy only (C/T), and 1 had undergone both radiotherapy and chemotherapy (RT and C/T).

The five key indicators of biofilm-associated wounds were assessed for all patients; see Table 2. Among the 10 cases, 4 patients met all 5 criteria, 3 patients met 5 out of 5, and 2 patients met 3 criteria. Delayed wound healing exceeding four weeks was documented in all cases. Exudate characteristics were classified as moderate in five cases and mild in five. Biofilm-like material was present in all cases, and wound cultures confirmed the presence of *Pseudomonas aeruginosa* in all patients. Antibiotic treatment failure was reported in seven patients. Signs of inflammation were observed in five patients. These findings support a high clinical suspicion of biofilm involvement in the included cases.

**Figure 1:**
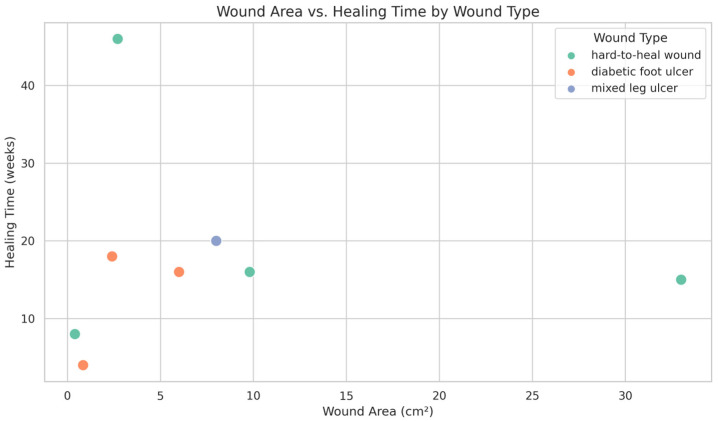
Wound Area and Healing Time Across Different Wound Types. This scatter plot illustrates the distribution of healing time (in weeks) in relation to wound area (cm^2^) among three wound types: hard-to-heal wounds (green), diabetic foot ulcers (orange), and mixed leg ulcers (blue). Each dot represents an individual patient case. This visualization highlights the diversity of wound characteristics and healing durations in a biofilm-associated wound cohort treated with medical-grade honey.

Reconstruction methods used included flap surgery in 2 cases and collagen dressing in 1 case, while the remaining 7 cases required no reconstruction in the end. The healing time following medical-grade honey (MGH) application was recorded for all patients. Among the 10 cases included, 8 achieved complete wound closure during the follow-up period. The median healing time was 16 weeks, ranging from 4 to 46 weeks, reflecting variability in wound severity and comorbidities (Figure 1). Notably, two cases did not achieve complete healing during the follow-up period. One was an 84-year-old female with a mixed leg ulcer who dropped out of outpatient follow-up. The other was a 46-year-old female with a breast cancer surgical wound ulcer who was undergoing radiotherapy and chemotherapy; she discontinued treatment due to the development of distal metastasis.

### Case Presentations

Case 1 (No. 1)

**Figure 2:**
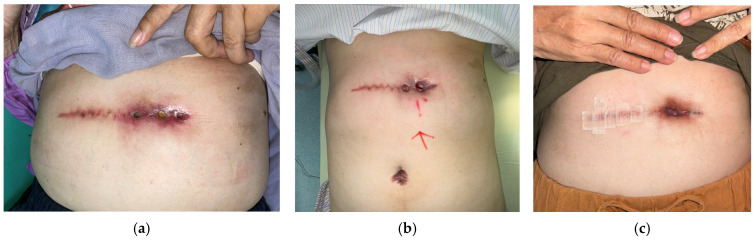
(**a**) A case of hypertrophic scar undergoing scar revision, presenting with a biofilm-associated abdominal ulcer persisting for weeks before MGH treatment; (**b**) The ulcer remained stable after 8 weeks of MGH therapy, (**c**) followed by uneventful healing 16 weeks after initiation of MGH therapy. The red arrow on (**b**) indicated the surgical site confirmation before surgery.

A 67-year-old female with a history of type II diabetes mellitus and gallbladder stones, status post-cholecystectomy, developed an abdominal ulcer measuring 7 × 1.4 cm following excision of a hypertrophic scar. Local erythema developed after a few days post-operatively (Figure 2a). Wound culture revealed *Achromobacter xylosoxidans* initially, while the follow-up culture yielded *Pseudomonas aeruginosa*. The ulcer presented with exudate, slough, and fibrin, with delayed healing despite four weeks of standard care and failed antibiotic therapy. Signs of inflammation, including periwound erythema and tenderness, were noted. MGH therapy was initiated, resulting in marked reduction in exudate and inflammation after 8 weeks (Figure 2b). A local flap procedure was performed to correct the undermined wound margin, and continued MGH application led to complete healing at week 16 (Figure 2c).

Case 2 (No. 3)

**Figure 3:**
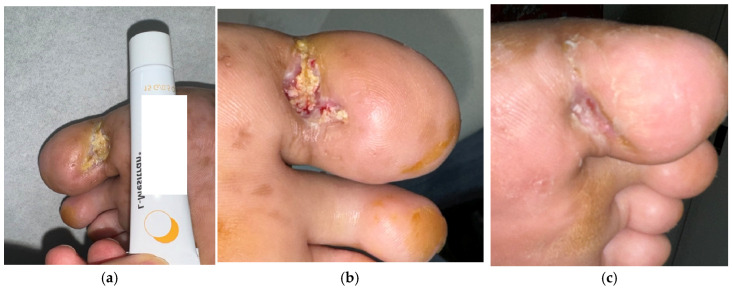
(**a**) A Pseudomonas-associated diabetic foot ulcer in a patient with diabetes and heart disease prior to MGH therapy; (**b**) showing improvement after 2 weeks of treatment and complete closure after 18 weeks (**c**).

A 33-year-old male with diabetes and underlying heart disease presented with a plantar ulcer over the left first toe, measuring 2 × 1.2 cm. The initial culture grew *Streptococcus agalactiae*, which was later followed by *Pseudomonas aeruginosa* on subsequent testing. He had a history of diabetic foot infection and multiple prior surgical debridements. Despite standard care and antibiotics, healing was delayed beyond four weeks. Clinical features included slough, fibrin, and mild exudate (Figure 3a). MGH therapy was initiated, and after two weeks the wound showed clear improvement (Figure 3b). Continued MGH treatment achieved complete healing after 18 weeks without further surgical intervention (Figure 3c).

Case 3 (No. 4)

**Figure 4:**
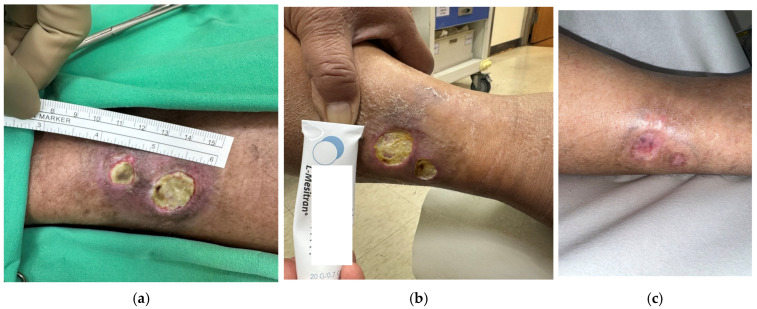
(**a**) A patient with oral cancer undergoing chemotherapy, presenting with a Pseudomonas-associated leg ulcer persisting for months; (**b**) The ulcer condition before initiation of MGH treatment; (**c**) Uneventful wound healing was achieved after surgical debridement with collagen dressing application.

A 56-year-old male with oral cancer undergoing chemotherapy presented with a chronic leg ulcer measuring 1.8 × 1.5 cm. The ulcer exhibited persistent inflammation, slough, fibrin, and moderate exudate, with no progression despite months of care and antibiotics (Figure 4a). Culture confirmed *Pseudomonas aeruginosa*. Surgical debridement was performed before initiating MGH therapy (Figure 4b). After 14 weeks of daily MGH application, the wound demonstrated notable improvement. Following a second debridement and subsequent application of collagen dressings, complete secondary healing was achieved 46 weeks after the initiation of MGH therapy (Figure 4c).

Case 4 (No. 5)

**Figure 5:**
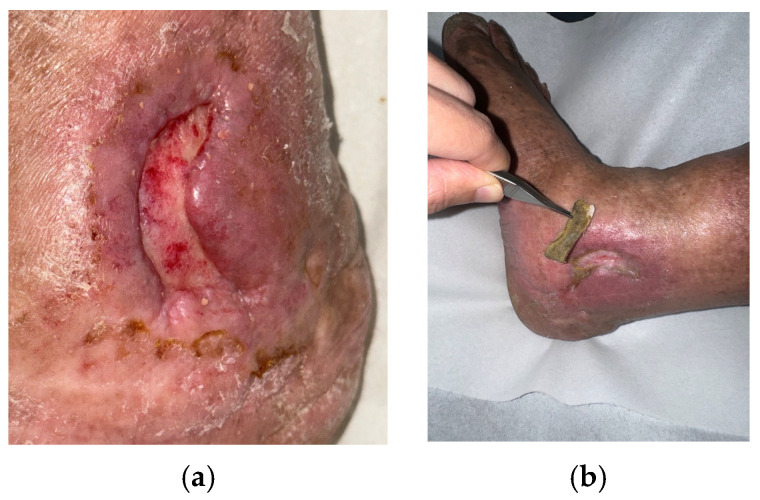
(**a**) A case of chronic venous insufficiency with a history of fasciotomy for right ankle fasciitis, presenting with a recurrent biofilm-associated leg ulcer before MGH therapy; (**b**) The ulcer improved after 8 weeks of treatment and achieved complete healing after 20 weeks.

A 64-year-old male with chronic venous insufficiency and prior right ankle fasciitis post-fasciotomy developed a recurrent ulcer measuring 4 × 2 cm. The ulcer showed fibrin, and poor healing despite four weeks of standard care (Figure 5a). Wound culture confirmed *Pseudomonas aeruginosa*. MGH therapy was started alongside standard care. The wound demonstrated marked reduction in exudate and resulted in complete closure after 20 weeks (Figure 5b).

Case 5 (No. 7)

**Figure 6:**
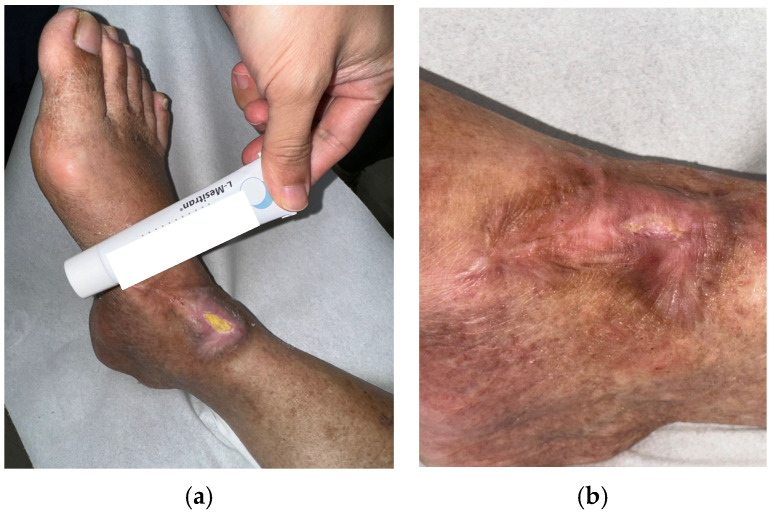
(**a**) A patient with diabetes mellitus, heart disease, peripheral arterial stenosis, and renal failure with uremia, presenting with a biofilm-associated right ankle ulcer persisting for weeks before MGH therapy; (**b**) The ulcer completely healed after 16 weeks of treatment.

A 55-year-old female with diabetes mellitus, heart disease, peripheral arterial stenosis, and renal failure with uremia presented with a right ankle ulcer measuring 3 × 2 cm. The wound bed contained slough and fibrin, with delayed healing and failed antibiotic therapy (Figure 6a). Culture confirmed *Pseudomonas aeruginosa*. MGH therapy was initiated with standard care, and after 16 weeks of treatment the ulcer achieved complete healing (Figure 6b).

Case 6 (No. 8)

**Figure 7:**
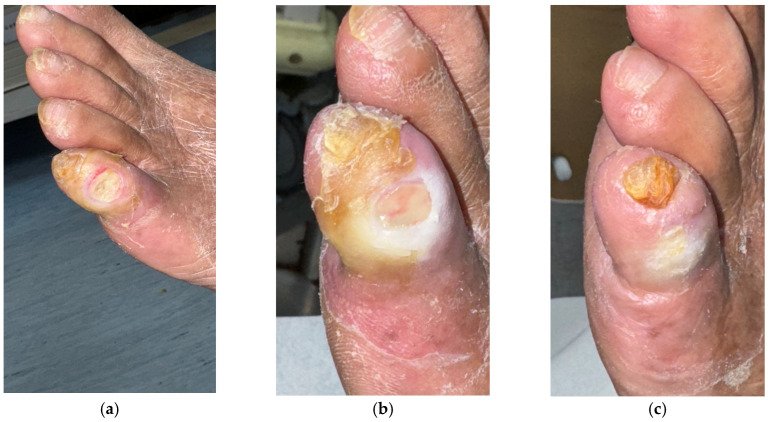
(**a**) Another patient with diabetes mellitus, heart disease, peripheral arterial stenosis, and renal failure with uremia, developed a biofilm-associated ulcer over the left fifth toe; (**b**) Osteomyelitis was excluded prior to MGH therapy; (**c**) The ulcer healed uneventfully after eight weeks of continuous MGH treatment.

A 76-year-old male with diabetes mellitus, heart disease, peripheral arterial stenosis, and renal failure with uremia developed an ulcer over the left fifth toe measuring 0.8 × 0.5 cm. The ulcer did not extend to the bone. The wound bed contained slough and fibrin, and despite standard care and antibiotic therapy, it failed to heal (Figure 7a). Wound culture confirmed *Pseudomonas aeruginosa*. MGH therapy was initiated following standard care (Figure 7b). The wound showed progressive improvement and achieved complete healing after eight weeks of continued MGH application (Figure 7c).

Case 7 (No. 9)

**Figure 8:**
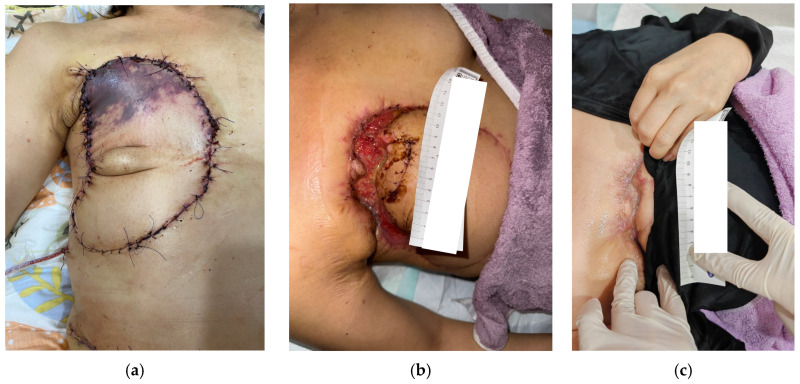
(**a**) A breast cancer patient following pedicled TRAM flap reconstruction developed tip ischemic necrosis at the right chest region; (**b**) A biofilm-associated wound was noted after surgical debridement with local flap coverage of the undermined edge before MGH therapy; (**c**) Uneventful healing was achieved after 15 weeks of MGH therapy.

A 46-year-old female with a history of breast cancer developed an ulcer measuring 11 × 3 cm over the right chest region following tip ischemic necrosis of a pedicle TRAM flap reconstruction (Figure 8a). The wound bed was covered with slough and fibrin and failed to heal despite four weeks of standard wound care and oral antibiotic therapy. Wound culture confirmed *Pseudomonas aeruginosa*. Surgical debridement with local flap coverage of the undermined ulcer edge was performed, followed by daily application of MGH for seven weeks, which resulted in progressive improvement and granulation tissue formation (Figure 8b). Subsequent skin grafting achieved complete and uneventful healing 15 weeks after the initiation of MGH therapy (Figure 8c).

## 3. Discussion

In this case series, MGH demonstrated a beneficial effect in reducing inflammation, controlling exudate, and enhancing healing in biofilm-associated wounds. Notably, most patients had comorbidities that could impair healing abilities, and many had failed prior systemic antibiotics. These findings align with the known properties of MGH in inhibiting microbial growth, disrupting established biofilms, and stimulating tissue repair [17,18,19].

Biofilm confirmation may be supported by advanced diagnostic tools, including Fluorescence imaging (e.g., MolecuLight) [20]. However, they are mostly unavailable in clinical settings due to the higher expenses, the longer turnaround time, and the feasibility of correlating clinical impressions with data. Therefore, clinical doctors use indicators such as persistent slough, fibrin, recurrent infection, and foul odor to help identify wounds with a high risk of biofilm.

Biofilm-associated bacteria often exhibit tolerance to systemic antibiotics due to their protective extracellular matrix and altered metabolic state. Even when culture-directed antibiotics are applied, eradication may be incomplete, leading to persistent inflammation and delayed healing. In these circumstances, wound improvement depends not only on antimicrobial therapy but also on mechanical debridement and topical agents capable of biofilm disruption—such as MGH. Therefore, biofilm-mediated tolerance may limit antibiotic effectiveness [2,21].

The management of biofilm-associated wounds requires both control and eradication of biofilm on the wound surface [22]. Mechanical debridement—sharp, autolytic, or enzymatic—is the first essential step to break down the biofilm matrix and reduce microbial burden [23]. Following debridement, topical agents with anti-biofilm properties, such as MGH, silver-based dressings, PHMB, or iodine preparations are recommended as adjunctive therapy [24,25]. In addition, surfactant-containing dressings can help break down the extracellular polymeric substances from biofilm [26]. Antibiofilm agents including xylitol, lactoferrin, or EDTA-based formulations offer disruption of microbial biofilm stability. Systemic agents such as doxycycline can be used not only for antimicrobial control but also for their anti-inflammatory and matrix-modulating properties [27,28].

Furthermore, adjuvant therapies such as negative pressure wound therapy (NPWT), photodynamic therapy, or low-frequency ultrasound have shown potential in enhancing wound bed preparation and accelerating biofilm clearance [29,30,31]. An integrated strategy that combines mechanical, chemical, and biological approaches is essential for optimizing outcomes in wounds complicated by biofilm.

The clinical outcomes observed in this case series align with existing evidence supporting the use of MGH in chronic and infected wounds with a high likelihood of biofilm presence. Previous studies have demonstrated that MGH facilitates autolytic debridement, reduces wound exudate, and enhances granulation tissue formation, which is consistent with the improvements noted in our patients [12,13,14,15,19]. Nair et al. reported that MGH successfully eradicated antibiotic-resistant bacteria and prevented lower-limb amputation in diabetics with infected ulcers in a prospective case series [13]. Papanikolaou et al. demonstrated favorable healing in clinically infected heel pressure ulcers among high-risk patients treated with MGH [14]. Similarly, Holubova et al. presented MGH as an effective alternative to systemic antibiotics in non-healing wounds, achieving bacterial control and progressive tissue repair [15].

Furthermore, the ability of MGH to suppress bacterial growth and disrupt biofilm has been reported in laboratories. Lu et al. confirmed that honey can effectively inhibit and eliminate *Pseudomonas aeruginosa* biofilms in vitro [17]. In addition, its immunomodulatory effects—including attenuation of excessive inflammation and stimulation of tissue repair pathways—have been described by Majtan et al., reinforcing the multifactorial mechanisms through which MGH contributes to wound recovery [18]. Collectively, these findings situate our results within the current body of literature and support MGH as an adjunct in the management of biofilm-associated wounds.

Our findings highlight that MGH offers distinct advantages in managing biofilm-suspected wounds. Its antimicrobial, anti-inflammatory, and autolytic debridement properties make it particularly effective in modifying the wound bed environment [12]. In cases with larger wound areas or slough-covered surfaces, debridement should be performed first, followed by the application of MGH to optimize the wound bed and reduce biofilm burden.

Once the wound bed is adequately prepared, reconstructive interventions—such as skin grafting or advanced dressing techniques—should be considered [32,33,34]. This combined approach has the potential to significantly accelerate healing, shorten the overall treatment period, and reduce the risk of complications such as infection, prolonged inflammation, or tissue necrosis. Together, these observations support a tiered treatment strategy: biofilm control and wound bed preparation using MGH, followed by timely reconstruction for wounds with delayed progression or extensive tissue loss.

Recent evidence from systematic reviews and meta-analyses further supports the clinical use of honey-based dressings. Yilmaz & Aygin reported that honey application may accelerate epithelialization and wound contraction in acute and chronic wounds [12]. A recent systematic review and meta-analysis by Karadeniz and Serin suggested that honey dressings may be associated with reduced wound healing time, increased healing rates, reduced pain, and shorter hospital stays in diabetic foot ulcer patients, although many included studies were small and heterogeneous, warranting cautious interpretation [35]. More recently, Tang et al. summarized current clinical data on honey for chronic wounds and concluded that while honey may improve healing outcomes, the certainty of evidence remains low due to heterogeneity and sample size limitations [36]. These findings align with the wound improvement trends observed in our case series, yet collectively emphasize that MGH should be interpreted as a supportive therapy rather than a definitive standalone solution.

This study has several limitations. First, the small sample size of only 10 cases limits the generalizability of the findings. Second, although clinical indicators strongly suggested the presence of biofilm, there was no definitive confirmation using advanced diagnostic methods such as confocal laser scanning microscopy or fluorescence in situ hybridization. Wounds of different etiologies—such as diabetic foot ulcers, hard-to-heal wounds, and mixed leg ulcers—present distinct physiological and pathological challenges that must be addressed individually [37,38,39]. For instance, diabetic foot ulcers often exhibit impaired microcirculation and neuropathy, while mixed leg ulcers are commonly associated with venous insufficiency and inflammatory edema. These inherent limitations influence not only the healing trajectory but also the wound’s responsiveness to treatment modalities.

Furthermore, due to the absence of a control group, this case series cannot determine whether MGH is superior to standard wound care. The observed wound improvements should therefore be interpreted as associative rather than causal. Nonetheless, these findings provide clinically relevant observations that may help inform the design of future studies. Larger investigations incorporating control groups or case–control designs are warranted to validate these preliminary results and to better define the role of MGH in the management of biofilm-associated wounds.

## 4. Materials and Methods

A retrospective review was conducted on 10 patients with biofilm-suspected wounds treated at KMUH and WS between August 2023 and November 2024. Data collected included demographic information, wound characteristics, photographic documentation, treatment modalities, and healing outcomes. Inclusion criteria required a clinical suspicion of biofilm presence and a lack of wound progression despite standard wound care after 4 weeks. Standard of care included comprehensive clinical assessment, wound evaluation, and optimization of systemic factors such as glycemic control, nutritional status, and vascular perfusion. Wound bed preparation followed the TIME principle, involving debridement of necrotic tissue as needed, infection and inflammation control with appropriate topical agents or systemic antibiotics when indicated, maintenance of a moist wound environment using suitable dressings according to exudate levels, and promotion of wound edge advancement.

Wound samples were obtained after gentle cleansing of the wound surface with sterile normal saline. Depending on wound depth and clinical condition, specimens were collected by swabbing using the Levine technique over an approximately 1 cm^2^ area of viable tissue with firm pressure.

Antibiotics were selected according to culture sensitivity results and modified if resistance patterns or clinical response indicated the need for an alternative agent. For cases with culture-proven *Pseudomonas aeruginosa*, systemic antimicrobial therapy was initiated with a combination of levofloxacin and doxycycline as the primary regimen.

Reconstructive surgery was performed at the discretion of the attending surgeon. MGH was continued as primary therapy in patients not requiring reconstruction and was also used as a preparatory wound bed optimization strategy when reconstruction was subsequently deemed beneficial.

Each case was evaluated for five key indicators of biofilm-associated wounds: signs of inflammation, delayed healing for more than four weeks, failure of antibiotic treatment, presence of biofilm-like material (slough, fibrin, necrosis), and wound culture data [40].

The medical-grade honey (MGH) used in this study was L-Mesitran^®^ Soft wound gel (Triticum Exploitatie BV, Maastricht, The Netherlands). This product is classified as medical-grade-honey-based on compliance with internationally accepted criteria for medical use, including: (1) controlled floral origin, (2) sterilization through gamma irradiation, (3) production under GMP-equivalent manufacturing standards, (4) proven antimicrobial and antioxidant bioactivity, and (5) safety certification conforming to medical device regulations. The definition and criteria for MGH were referenced according to Peters et al. [11].

MGH was applied topically and directly to the wound bed as a thin film layer, ensuring full coverage of the wound surface. A secondary dressing was placed to maintain moisture balance and protect the wound with dressing changes mainly performed once daily and adjusted based on exudate amount. Outcome measures included wound healing status and time to complete healing.

Patient age and follow-up time are reported as median and range to better reflect the distribution and wound size is reported as mean ± standard error of the mean (SEM) to account for the small sample size.

Generative artificial intelligence (GenAI) tools, specifically ChatGPT (GPT-4o version, OpenAI, San Francisco, CA, USA), were used to assist in the refinement of English grammar, sentence structure, and academic tone of the manuscript. The clinical content, data interpretation, and conclusions were conceived, analyzed, and verified by the authors. AI tools were used to analyze data, and Figure 1. All clinical content and interpretations were authored and verified by the investigators.

## 5. Conclusions

This retrospective case series suggests that medical-grade honey (MGH) may represent a beneficial topical option for wounds complicated by biofilm. The use of MGH was associated with favorable changes in wound healing parameters, including reduction of exudate and local inflammation, as well as progression toward wound closure in some cases without the need for immediate surgical intervention. In selected complex wounds, MGH was also applied as an adjunctive therapy and appeared to improve wound bed conditions, thereby facilitating subsequent reconstructive procedures. This potential dual role of MGH—as both a topical antimicrobial dressing and a preparatory modality for surgical closure—highlights its versatility in the management of hard-to-heal, biofilm-associated wounds. However, larger prospective studies and randomized controlled trials are required to validate these observations, establish standardized treatment protocols, and further define the role of MGH within integrated wound care strategies.

## Figures and Tables

**Table 1:** Patient profiles.

Case No.	Sex	Age (Year)	Wound Classification	Wound Age (Weeks)	Wound Site	Underlying Dx	RT or C/T	Wound Length (cm)	Wound Width (cm)	Wound Area (cm^2^)
1	Female	67	Hard-to-heal wound	4	Abdomen	Liver disease, dm	Nil	7.00	1.40	9.80
2	Male	51	Diabetic foot ulcer	4	Ankle	DM	Nil	1.20	0.70	0.84
3	Male	33	Diabetic foot ulcer	20	Foot	DM, heart disease	Nil	2.00	1.20	2.40
4	Male	56	Hard-to-heal wound	n/a	Leg	Oral cancer	C/T	1.80	1.50	2.70
5	Male	64	Mixed leg ulcer	2	Ankle	Chronic venous insufficiency	Nil	4.00	2.00	8.00
6	Female	84	Mixed leg ulcer	12	Leg	PAD, bilateral iliac artery stenosis, chronic venous insufficiency	Nil	3.00	2.00	6.00
7	Female	55	Diabetic foot ulcer	4	Ankle	DM, heart disease, PAD, uremia	Nil	3.00	2.00	6.00
8	Male	76	Hard-to-heal wound	2	Toe	DM, heart disease, PAD, uremia	Nil	0.80	0.50	0.40
9	Female	60	Hard-to-heal wound	11	Breast	Breast cancer	C/T	11.00	3.00	33.00
10	Female	46	Hard-to-heal wound	4	Breast	Breast cancer	RT and C/T	4.50	1.20	5.40
**Case No.**	**Wound Etiology**		**Previous Treatments**	**Identified Pathogenic Bacteria**		**Reconstruction Method**	**Exudate Characteristics**		**Wound Healed After MGH Use (Week)**	
1	Surgical site infection		Debridements	*Achromobacter xylosoxidans/Pseudomonas aeruginosa*		Local flap	Moderate		16	
2	Diabetic foot infection		SoC	*Pseudomonas aeruginosa*		Nil	Mild		4	
3	Diabetic foot infection		Serial debridements and skin graft	*Streptococcus agalactiae/Pseudomonas aeruginosa*		Nil	Mild		18	
4	Trauma		SoC	*Pseudomonas aeruginosa*		Collagen dressing	Moderate		46	
5	Venous ulcer		SoC	*Pseudomonas aeruginosa*		Nil	Moderate		20	
6	Ischemic ulcer		Debridements	*Pseudomonas aeruginosa*		Nil	Moderate		n/a	
7	Diabetic foot infection		SoC	*Finegoldia magna/Pseudomonas aeruginosa*		Nil	Mild		16	
8	Ischemic ulcer		Serial debridements	*Pseudomonas aeruginosa*		Nil	Mild		8	
9	Flap necrosis		Serial debridements	*Pseudomonas aeruginosa*		Debridement, local flap and skin graft	Moderate		15	
10	Wound dehiscence after chemotherapy		SoC	*Pseudomonas aeruginosa*		n/a	Mild		n/a	
**Case No.**	**Follow-Up Time (Months)**			**Complications or Recurrence**			**Remarks**			
1	24			No			Nil			
2	20			No			Nil			
3	20			No			Another episode of infection occurred 15 months after wound healed			
4	18			No			Nil			
5	20			No			Nil			
6	26			No			Unhealed wound			
7	20			No			Nil			
8	20			No			Nil			
9	11			No			Cancer progression accompanied by with skin metastasis			
10	8			No			Unhealed wound			

Abbreviations: SoC, standard of care; n/a, not available; DM, diabetes mellitus; C/T, chemotherapy; RT, radiation therapy; PAD, peripheral arterial disease.

**Table 2:** Case profiles of biofilm-associated wound criteria.

Case No.	Inflammation Signs	Delayed Healing > 4 Weeks	Failure of Antibiotic Treatment	Presence of Biofilm-like Material (Slough, Fibrin, Necrosis)	Wound Culture Detection
1	Yes	Yes	Yes	Yes	Yes
2	No	Yes	No	Yes	Yes
3	No	Yes	Yes	Yes	Yes
4	Yes	Yes	Yes	Yes	Yes
5	No	Yes	No	Yes	Yes
6	Yes	Yes	Yes	Yes	Yes
7	No	Yes	Yes	Yes	Yes
8	No	Yes	Yes	Yes	Yes
9	Yes	Yes	Yes	Yes	Yes
10	Yes	Yes	No	Yes	No

## Data Availability

The data that support the findings of this study are available from the corresponding author upon reasonable request. All data relevant to the study are included in the article.

## Abbreviations

The following abbreviations are used in this manuscript:

MGH	Medical-grade honey
DOAJ	Methicillin-resistant Staphylococcus aureus
RT	Radiotherapy
C/T	Chemotherapy
PAD	Peripheral arterial disease
DM	Diabetes mellitus
SEM	Scanning electron microscopy
NPWT	Negative pressure wound therapy
PHMB	Polyhexamethylene biguanide
EDTA	Ethylenediaminetetraacetic acid
GenAI	Generative artificial intelligence
SEM	Standard error of the mean
